# Characterization of the complete mitochondrial genome of *Brentisentisyangtzensis* Yu & Wu, 1989 (Acanthocephala, Illiosentidae)

**DOI:** 10.3897/zookeys.861.34809

**Published:** 2019-07-08

**Authors:** Rui Song, Dong Zhang, Jin-Wei Gao, Xiao-Fei Cheng, Min Xie, Hong Li, Yuan-An Wu

**Affiliations:** 1 Hunan Fisheries Science Institute, Changsha 410153, China Hunan Fisheries Science Institute Changsha China; 2 Collaborative Innovation Center for Efficient and Health Production of Fisheries in Hunan Province, Changde, 415000, China Collaborative Innovation Center for Efficient and Health Production of Fisheries in Hunan Province Changde China; 3 Key Laboratory of Aquaculture Disease Control, Ministry of Agriculture, and State Key Laboratory of Freshwater Ecology and Biotechnology, Institute of Hydrobiology, Chinese Academy of Sciences, Wuhan 430072, China Institute of Hydrobiology, Chinese Academy of Sciences Wuhan China

**Keywords:** Echinorhynchida, gene order, molecular phylogeny

## Abstract

The mitogenome of *Brentisentisyangtzensis* is 13,864 bp in length and has the circular structure typical of metazoans. It contains 36 genes: 22 transfer RNA genes (tRNAs), two ribosomal RNA genes (rRNAs) and 12 protein-encoding genes (PCGs). All genes are transcribed from the same strand. Thirteen overlapping regions were found in the mitochondrial genome. The overall A+T content of *B.yangtzensis* is 68.3% versus 31.7% of G+C content (A = 27.8%, T = 40.5%, C = 9.0%, G = 22.7%). *B.yangtzenensis* (Illiosentidae) and *Leptorhynchoidesthecatus* (Rhadinorhynchidae) form a sister clade, showing the relatively close relationship between the Illiosentidae and the Rhadinorhynchidae. The mitochondrial gene arrangements of acanthocephalan species are relatively conserved, with only a few translocations of tRNAs (trnS1, trnS2, trnV, and trnK) detected. An identical gene order was found both in a sister clade (*Centrorhynchusaluconis* and *Plagiorhynchustransversus*) and across different classes (*B.yangtzensis* (Palaeacanthocephala), *Acanthosentischeni* (Eoacanthocephala) and *Macracanthorhynchushirudinaceus* (Archiacanthocephala), *Oncicolaluehei* and *L.thecatus* (Palaeacanthocephala)). More studies and more sequences of acanthocephalan species are needed to gain a clear understanding of the phylogenetic relationships.

## Introduction

Members of the Acanthocephala are obligate endoparasites which utilize arthropods as intermediate hosts and vertebrates as definitive hosts. This phylum contains approximately 1300 documented species, and is classified into three classes (Archiacanthocephala, Palaeacanthocephala and Eoacanthocephala). The Palaeacanthocephala has the highest species richness with 65% of the total acanthocephalan species, and comprises three orders: Echinorhynchida, 472 species; Polymorphida, 372 species; and Heteramorphida, one species (Amin 1987, 2013). The classifications proposed by Golvan (1960) and Amin (1987) have been challenged by recent phylogenetic studies, which indicated that the genus *Leptorhynchoides* (Rhadinorhynchidae Kostylew, 1924) is more closely related to the genera of the Illiosentidae Golvan, 1960 rather than those of the Rhadinorhynchidae in morphological (Monks 2001) and the molecular phylogenies (García-Varela and Nadler 2005; García-Varela and González-Oliver 2008). Intriguingly, *Illiosentis* Van Cleave & Lincicome, 1939 was first placed in the Rhadinorhynchidae (Van Cleave and Lincicome 1939), but Golvan (1960) decided that a new family was required to accommodate the genus, and so erected the Illiosentidae.

This debate continues, and molecular markers carrying stronger phylogenetic signals are needed to resolve the phylogenetic relationships with a higher resolution. The mitogenome is a good candidate, being approximately ten times larger than commonly used single-gene molecular markers (ITS, 18S, and 28S) (Zhang et al. 2018), and considered to provide the best interrelationship estimate for the Cestoda (Waeschenbach et al. 2012). Mitochondrial genome sequences are becoming prevalent and are increasingly used in population genetics (Yin et al. 2015), phylogenetics (Gazi et al. 2012, 2015, 2016; Weber et al. 2013; Li et al. 2017; Pan and Nie 2013) and the diagnostics (Huyse et al. 2008; Jia et al. 2010) of metazoans. However, many groups of parasitic organisms are unrepresented, and the resolving power of mitochondrial genomics is still limited by the small number of acanthocephalan mitogenomes (only 13 species available), with many taxonomic categories, e.g. the Illiosentidae, poorly represented or unrepresented.

The complete mitogenome of an illiosentid species has not previously been published. In order to fill this knowledge gap, we have sequenced and annotated the complete mitogenome of *Brentisentisyangtzensis* Yu & Wu, 1989 (Palaeacanthocephala, Illiosentidae), a parasite from the intestines of many freshwater fish species in the middle reaches of the Yangtze River (Yi and Wu 1989). Previous studies on this parasite have focused on its morphology and population ecology (Fang 1999, Fang and Dai 2000; Fang et al. 2004); molecular data have not previously been reported.

## Materials and methods

### Specimen collection and DNA extraction

The acanthocephalans were collected on 24 September 2018 from the intestine of 36 bullhead catfish *Tachysurusfulvidraco* (Richardson, 1846) from east Dongting Lake in Yueyang (29°22'N, 113°06'E), Hunan Province, China. *Brentisentisyangtzensis* was identified by morphology (e.g., Yu 1989) using a stereomicroscope and a light microscope. The parasites were preserved in 100% ethanol and stored at 4 °C. The total genomic DNA was extracted from an entire acanthocephalan using a TIANamp Micro DNA Kit (Tiangen Biotech, Beijing, China) according to manufacturer’s recommended protocol, and stored at -20 °C. Eleven acanthocephalans were collected in total.

### PCR and DNA sequencing

Partial sequences of rrnL, cytb, nad1, and rrnS genes were amplified via a polymerase chain reaction (PCR) using four primer pairs. Based on these fragments, we designed specific primers for subsequent PCR amplification (Suppl. material 1). PCR reactions were conducted in a 50 ml reaction mixture, containing 18.5 ml double-distilled water (dd H_2_O), 25 ml 2×PCR buffer (Mg^2+^, dNTP plus, Takara, China), 1.5 ml of each primer, 1 ml rTaq polymerase (250U, Takara, China) and 2.5 ml DNA template. Amplification was performed under the following conditions: initial denaturation at 98 °C for 2 min, followed by 40 cycles at 98 °C for 10 s, 48–60 °C for 15 s, 68 °C for 1 min/kb, and a final extension at 68 °C for 10 min. PCR products were sequenced bidirectionally at Sangon Biotech (Shanghai) Co., Ltd. (China) using the primer walking strategy.

### Sequence annotation analyses

The mitogenome of *B.yangtzensis* were assembled manually in a stepwise manner with the help of the DNAstar v7.1 program (Burland 2000), after quality-proofing of the obtained fragment. The mitogenome was annotated mainly following the procedures described previously (Zou et al. 2017; Zhang et al. 2017a; Li et al. 2017). In detail, protein-coding genes (PCGs) were inferred with the help of BLAST and ORF Finder tools (both available from the National Center for Biotechnology Information (NCBI)), employing the invertebrate mitochondrial code (Codon Table 5), and checking the nucleotide alignments against the reference genomes in acanthocephalan *Leptorhynchoidesthecatus* (Linton, 1891) Kostylew, 1924 (NC_006892). A majority of the tRNAs were identified using the results of ARWEN (Laslett and Canback 2008) and MITOS web server (Bernt et al. 2013), the rest were found by alignment with other acanthocephalans (Suppl. material 2). Two ribosomal RNA genes (rrnL and rrnS) were found by alignment with other published acanthocephalan mitogenomes, and their ends were assumed to extend to the boundaries of their flanking genes. Codon usage and relative synonymous codon usage (RSCU) for 12 protein-encoding genes (PCGs) of the *B.yangtzensis* and *L.thecatus* (NC_006892) were computed and sorted using PhyloSuite (Zhang et al. 2018), and finally the RSCU figure drawn using ggplot2 plugin (Wickham 2016). The circular map of *B.yangtzensis* mitogenome was drawn with the mitochondrial visualization tool MTVIZ (http://pacosy.informatik.uni-leipzig.de/mtviz/).

### Phylogenetic analyses

Phylogenetic analyses were carried out on the newly sequenced mitogenome of *B.yangtzensis* and the 12 acanthocephalan mitogenomes available in GenBank (Suppl. Table S2). Two species of the Bdelloidea, *Rotariarotatoria* (Pallas, 1766) (NC013568.1) and *Philodinacitrina* Ehrenberg, 1832 (FR856884.1), were used as outgroups. Fasta files with the amino acid sequences for all 12 PCGs were extracted from the GenBank files using PhyloSuite. All the genes were aligned in batches with MAFFT (Katoh et al. 2002) integrated in PhyloSuite, using normal-alignment mode. PhyloSuite was then used to concatenate these alignments into a single alignment and generate phylip and nexus format files for the phylogenetic analyses, conducted using maximum likelihood (ML) and Bayesian inference (BI) methods. The selection of the best-fit partition strategy and models was carried out using PartitionFinder2 (Lanfear et al. 2017). ML analysis was performed using IQ-TREE (Nguyen et al. 2015) with 50, 000 Ultrafast bootstraps (Minh et al. 2013). BI analysis was performed in MrBayes 3.2.6 (Ronquist et al. 2012) with the default settings, and 3 × 10^6^ metropolis-coupled Markov Chain Monte Carlo generations.

## Results and discussion

### Genome organization and base composition

**Figure 1.**
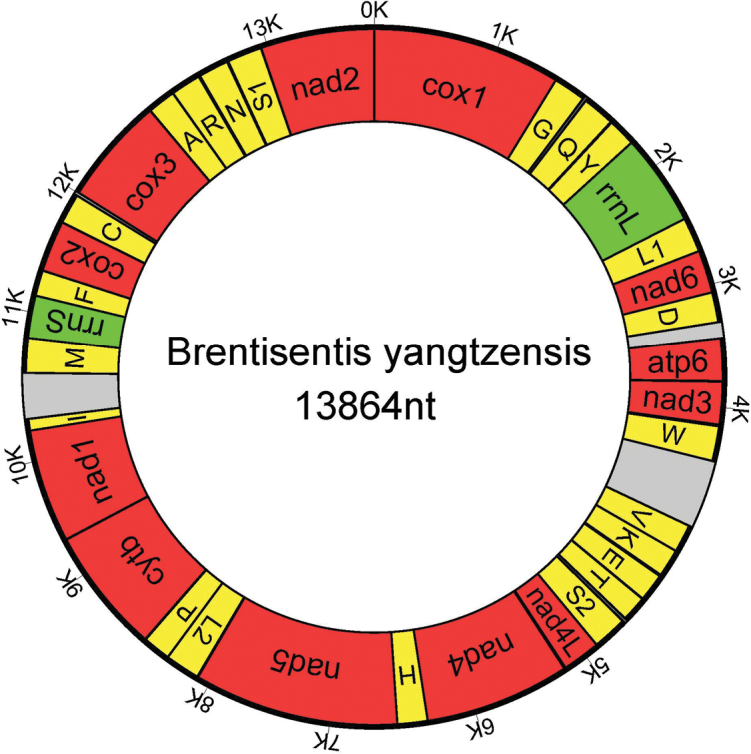
Map of the complete mitochondrial genome of *Brentisentisyangtzensis*.

The circular duplex molecule mitogenome of *B.yangtzensis* is 13,864bp in size (GenBank accession number MK651258) and contains all 36 of the typical metazoan genes: 22 tRNA genes, 2 rRNA genes and 12 protein-encoding genes (PCGs) (lacking atp8) (Fig. 1). All genes are transcribed from the same strand, and 13 overlapping regions were found in the genome (Table 1). The lack of the gene atp8 is common in acanthocephalans (Gazi et al. 2016; Song et al. 2016) with one exception; in *L.thecatus* two putative atp8 genes have been suggested (Steinauer et al. 2005).

**Table 1.** Annotated mitochondrial genome of *Brentisentisyangtzensis*.

Gene	Position		Size	Intergenic nucleotides	Codon		Anti-codon
	From	To			Start	Stop	
cox1	1	1531	1531	–	GTG	T	–
trnG	1532	1585	54	–	–	–	TCC
trnQ	1565	1630	66	-21	–	–	TTG
trnY	1626	1678	53	-5	–	–	GTA
rrnL	1679	2590	912	–	–	–	–
trnL1	2591	2644	54	–	–	–	TAG
nad6	2645	3080	436	–	GTG	T	–
trnD	3081	3135	55	–	–	–	GTC
atp6	3240	3797	558	104	ATG	TAG	–
nad3	3794	4147	354	-4	ATA	TAG	–
trnW	4138	4197	60	-10	–	–	TCA
trnV	4635	4694	60	437	–	–	TAC
trnK	4695	4755	61	–	–	–	CTT
trnE	4747	4800	54	-9	–	–	TTC
trnT	4803	4872	70	2	–	–	TGT
trnS2	4851	4900	50	-22	–	–	TGA
nad4L	4901	5149	249	–	ATG	TAA	–
nad4	5159	6413	1255	9	GTG	T	–
trnH	6414	6466	53	–	–	–	GTG
nad5	6467	8110	1644	–	ATG	TAG	–
trnL2	8106	8159	54	-5	–	–	TAA
trnP	8160	8211	52	–	–	–	TGG
cytb	8215	9346	1132	3	ATG	T	–
nad1	9345	10242	898	-2	TTG	T	–
trnI	10243	10301	59	–	–	–	GAT
trnM	10595	10651	57	293	–	–	CAT
rrnS	10652	11225	574	–	–	–	–
trnF	11226	11281	56	–	–	–	GAA
cox2	11281	11931	651	-1	GTG	TAG	–
trnC	11931	11983	53	-1	–	–	GCA
cox3	12005	12736	732	21	ATG	TAG	–
trnA	12736	12791	56	-1	–	–	TGC
trnR	12793	12854	62	1	–	–	TCG
trnN	12846	12900	55	-9	–	–	GTT
trnS1	12894	12946	53	-7	–	–	ACT
nad2	12949	13863	915	2	ATG	TAG	–

### Protein-coding genes and codon usage

The total length of the concatenated 12 protein-coding genes is 10,355 bp, with the average A+T content of 68.0%, ranging from 65.9% (nad3) to 69.5% (atp6 and nad4) (Suppl. material 3). ATG (for 6 PCGs) is the most commonly used start codon, whereas nad6, nad4, cox1 and cox2 used GTG, nad1 and nad3 used TTG and ATA, respectively. The most frequent terminal codons are TAG (for 7 PCGs), followed by T (4 PCGs) (Table 1).

Codon usage, relative synonymous codon usage (RSCU) and codon family proportion (corresponding to the amino acids usage) of *B.yangtzensis* and *L.thecatus* (NC_006892) is presented (Suppl. material 4). Leucine (16.28%), valine (11.92%) and serine (10.94%) are the most frequent amino acids in the PCGs of *B.yangtzensis*, whereas glutamine (0.81%), arginine (1.36%) and histidine (1.44%) are relatively scarce. A higher T content (42.2%) in 12 PCGs corresponds to a relatively high frequency of T-rich codons: TTA for leucine (9.9%), TTT for phenylalanine (7.2%), ATT for isoleucine (6.1%) and GTT for valine (5.0%).

### Transfer and ribosomal RNA genes

All 22 commonly found tRNAs are present in the mitogenome of *B.yangtzensis*, ranging from 50 bp (trnS2) to 70 bp (trnT) in size, with a concatenated length of 1,247 bps (Table 1).

**Figure 2.**
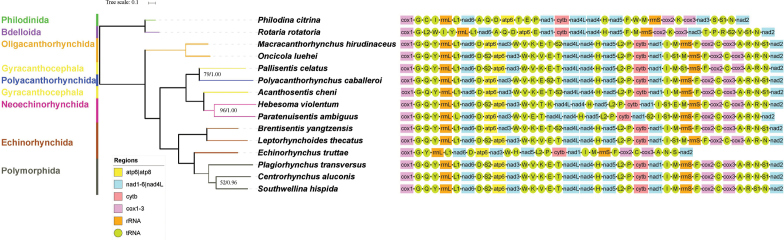
Phylogenetic tree of acanthocephalans inferred from maximum likelihood analysis with concatenated nucleotide sequence of all 36 genes (12 PCGs, 2 rRNAs, and 22 tRNAs). Bootstrap (BS)/Bayesian posterior probability (BPP) support values are shown above the nodes, only BS < 100 and BPP < 1 are displayed.

The genes rrnL and rrnS are 912 bp and 574 bp in size, with 71.8% and 69.8% A+T content, respectively (Suppl. Material 3). The location of rrnL is between trnY and trnL1, and rrnS is located between trnM and trnF; this is the same arrangement reported for other acanthocephalans (Fig. 2), except for *Hebesomaviolentum* (Van Cleave, 1928) Salgado-Maldonado, 1978 in which rrnS is located between trnS1 and trnF (Pan and Nie 2014).

### Phylogeny

BI and ML yielded phylograms with identical topology and strong statistical support for all nodes (BP ≥ 75, BPP ≥ 0.96) (except the branch of *Centrorhynchusaluconis* (Müller, 1780) Lühe 1911 and *Southwellinahispida* (Van Cleave, 1925) Witenberg, 1932 52/0.96). Since both phylograms have an identical topology, only the latter was shown (Fig. 2). The resulting combined phylogenetic tree depicted almost the same results found in previous mitogenomic studies (Gazi et al. 2016), except for the position of *B.yangtzensis* (Palaeacanthocephala, Illiosentidae), as this was included for the first time in the present study. Within the Acanthocephala, tree topology indicates the existence of two major clades: class Archiacanthocephala (monophyletic and the most basal clade) and the other two classes (Eoacanthocephala + Palaeacanthocephala), resulting in the three monophyletic clades corresponding to the three classes (Archiacanthocephala, Eoacanthocephala, and Palaeacanthocephala) in the most widely accepted classification of the Acanthocephala.

The Echinorhynchida is paraphyletic, with three species separated into two clades: *Echinorhynchustruttae* Schrank, 1788 (Echinorhynchidae) formed a sister clade with species of the Polymorphida and *B.yangtzensis* (Illiosentidae) formed a sister clade with *L.thecatus* (Rhadinorhynchidae). The result shows the relatively close relationship between the Illiosentidae and the Rhadinorhynchidae; however, as each family of the Echinorhynchida was represented by a single species in our study, this topology should be interpreted with some caution. Previous studies have shown the close relationship between *Leptorhynchoides* (Rhadinorhynchidae) and a genus of the Illiosentidae (Monks 2001; García-Varela and Nadler 2005; García-Varela and González-Oliver 2008). Two species of the Gyracanthocephala Van Cleave, 1936, one (*Pallisentiscelatus* (Van Cleave, 1928) Baylis, 1933) formed a sister clade with *Polyacanthorhynchuscaballeroi* Diaz-Ungria et Rodrigo, 1960 (Polyacanthorhynchida Amin, 1987), and another (*Acanthosentischeni* Amin, 2005) nested with species of the Neoechinorhynchida. This result suggests paraphyly of the Gyracanthocephala and corresponds with previous phylogenetic analyses (Song et al. 2016). As the lack of the mitochondrial genome information on acanthocephalan species, more studies and sequences of acanthocephalan (Illiosentidae and Rhadinorhynchidae) species are needed to gain a clear understanding of the phylogenetic relationships of these acanthocephalans.

### Gene order

The mitochondrial gene arrangements of acanthocephalan species are relatively conserved (Fig. 2). Besides the incomplete mitochondrial genome of *E.truttae*, the gene arrangement of 12 protein coding genes and two rRNA genes are highly conserved, with only a few translocations of tRNAs (trnS1, trnS2, trnV, and trnK) detected. In mitochondrial genomes, conserved gene arrangement is considered to be a typical characteristic (Boore 2000; Li et al. 2012), but the arrangement of tRNAs shows more variability, and there are examples of extensive gene rearrangement (Hyman et al. 2011; Zhang et al. 2017b; Wang et al. 2017). Transfer RNA genes are more often translocated than other genes, probably because of their small size (Kilpert and Podsiadlowski 2006).

In many cases, the gene order of the mitochondrial genome can form useful information in inferring phylogenetic relationships of metazoans (Littlewood et al. 2006; Luo et al. 2008; Wang et al. 2017; Zhang et al. 2018); however, for the taxa in our study, a phylogeny inferred from gene order is incompatible with that based on amino acid sequence (Fig. 2). The translocations of four tRNAs (trnS2, trnV, trnk, trnS1) were detected between *B.yangtzensis* and *L.thecatus* (Echinorhynchida), which formed a monophyletic clade in the phylogenetic tree. However, only 1–4 translocations of tRNAs were detected among all of the acanthocephalan species. And these four tRNA (trnS2, trnV, trnk, trnS1) translocations were also detected within the Archiacanthocephala clade. Moreover, identical gene order was found both in a sister clade (*C.aluconis* and *Plagiorhynchustransversus* (Rudolphi, 1819) Travassos, 1926) and across different classes (*B.yangtzenensis* (Palaeacanthocephala), *A.cheni* (Eoacanthocephala), and *Macracanthorhynchushirudinaceus* (Pallas, 1781) Travassos, 1917 (Archiacanthocephala); and *Oncicolaluehei* (Travassos, 1917) Schmidt, 1972 (Archiacanthocephala) and *L.thecatus* (Palaeacanthocephala)).

## Supplementary materials

Supplementary material 1 Table S1. Primers used to amplify and sequence the mitochondrial genomes of *Brentisentisyangtzensis* Data type: molecular data https://binary.pensoft.net/file/315158This dataset is made available under the Open Database License (http://opendatacommons.org/licenses/odbl/1.0/). The Open Database License (ODbL) is a license agreement intended to allow users to freely share, modify, and use this Dataset while maintaining this same freedom for others, provided that the original source and author(s) are credited.Rui Song, Dong Zhang, Jin-Wei Gao, Xiao-Fei Cheng, Min Xie, Hong Li, Yuan-An Wu

Supplementary material 2 Table S2. The list of species of the Acanthocephala and the outgroups used for comparative mitogenomic and phylogenetic analyses Data type: species data https://binary.pensoft.net/file/315159This dataset is made available under the Open Database License (http://opendatacommons.org/licenses/odbl/1.0/). The Open Database License (ODbL) is a license agreement intended to allow users to freely share, modify, and use this Dataset while maintaining this same freedom for others, provided that the original source and author(s) are credited.Rui Song, Dong Zhang, Jin-Wei Gao, Xiao-Fei Cheng, Min Xie, Hong Li, Yuan-An Wu

Supplementary material 3 Table S3, Nucleotide composition and skewness of different elements of the studied mitochondrial genome Data type: molecular data https://binary.pensoft.net/file/315160This dataset is made available under the Open Database License (http://opendatacommons.org/licenses/odbl/1.0/). The Open Database License (ODbL) is a license agreement intended to allow users to freely share, modify, and use this Dataset while maintaining this same freedom for others, provided that the original source and author(s) are credited.Rui Song, Dong Zhang, Jin-Wei Gao, Xiao-Fei Cheng, Min Xie, Hong Li, Yuan-An Wu

Supplementary material 4 Figure S1. Relative Synonymous Codon Usage (RSCU) of *Brentisentisyangtzensis* Data type: molecular data https://binary.pensoft.net/file/315161This dataset is made available under the Open Database License (http://opendatacommons.org/licenses/odbl/1.0/). The Open Database License (ODbL) is a license agreement intended to allow users to freely share, modify, and use this Dataset while maintaining this same freedom for others, provided that the original source and author(s) are credited.Rui Song, Dong Zhang, Jin-Wei Gao, Xiao-Fei Cheng, Min Xie, Hong Li, Yuan-An Wu
