# Supplementary figures and images for: Characterization of the complete mitochondrial genome of Brentisentisyangtzensis Yu & Wu, 1989 (Acanthocephala, Illiosentidae)
Source: Zookeys. 2019 Jul 8;861:1–14. doi: 10.3897/zookeys.861.34809 (PMC6656981; doi:10.3897/zookeys.861.34809)

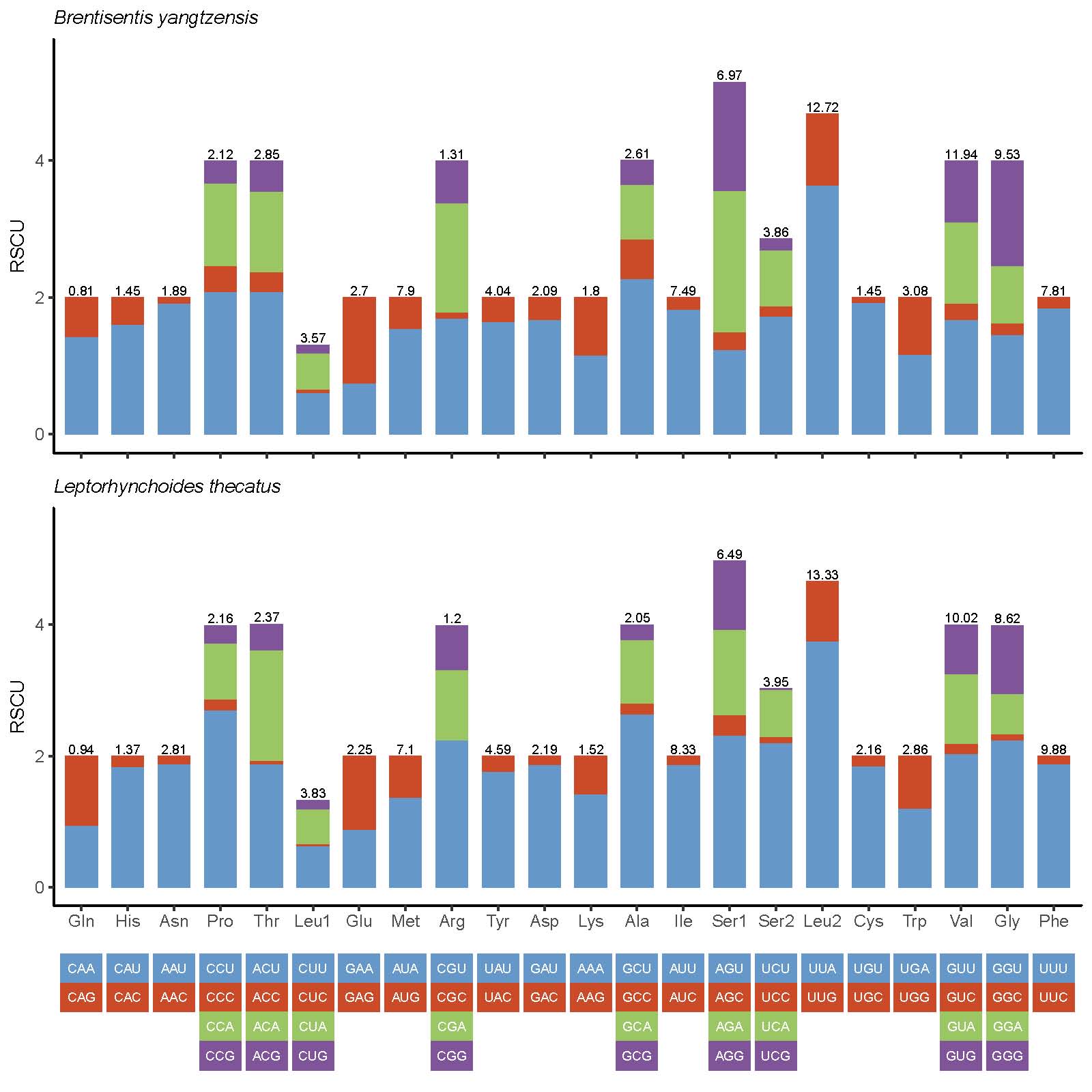

Supplement: Supplementary material 4 [file zookeys-861-001-s004.jpg]
